# Intercalation-assisted longitudinal unzipping of carbon nanotubes for green and scalable synthesis of graphene nanoribbons

**DOI:** 10.1038/srep22755

**Published:** 2016-03-07

**Authors:** Yan-Sheng Li, Jia-Liang Liao, Shan-Yu Wang, Wei-Hung Chiang

**Affiliations:** 1 Department of Chemical Engineering, National Taiwan University of Science and Technology, Taipei, 10607, Taiwan

## Abstract

We have demonstrated an effective intercalation of multi-walled carbon nanotubes (MWCNTs) for the green and scalable synthesis of graphene nanoribbons (GNRs) using an intercalation-assisted longitudinal unzipping of MWCNTs. The key step is to introduce an intercalation treatment of raw MWCNTs with KNO_3_ and H_2_SO_4_, making it promising to decrease the strong van der Waals attractions in the MWCNTs bundles and between the coaxial graphene walls of CNTs. Systematic micro Raman, X-ray photoelectron spectroscopy (XPS), and X-ray diffraction (XRD) characterizations suggest that potassium, nitrate, and sulfate ions play an important role in the CNT intertube and intratube intercalations during the pretreatment. Detailed scanning electron microscopy (SEM), transmission electron microscopy, XRD, and micro Raman characterizations indicate that the developed methodology possesses the ability to synthesis GNRs effectively with an improved CNT concentration in H_2_SO_4_ of 10 mg/ml at 70 °C, which is amenable to industrial-scale production because of the decreased amount of strong acid. Our work provides a scientific understanding how to enhance the GNR formation by accelerating the CNT longitudinal unzipping via suitable molecular intercalation.

Graphene nanoribbons (GNRs) represent a unique form of carbon materials and have spurred intensive interests due to their exceptional physical and chemical properties^1^,^2^,^3^. Recent reports suggest that GNRs are superior materials for many applications such as energy generation and storage^4^,^5^, chemical and biosensors^5^,^6^, catalysis^7^,^8^, nanocomposites^9^,^10^, and nanoelectronics^11^,^12^. Despite their advantages for many applications, it is still challenging to synthesis GNRs efficiently in a bulk quantity^13^. Consequently the development of a facile, large-scale, and high-yield production of GNRs will lead to important advances on both fundamental study and innovative applications.

Many methods including longitudinal unzipping of carbon nanotubes (CNTs)^14^, plasma etching^15^, metal catalyst assisted cutting^16^,^17^, lithium insertion and exfoliation^18^,^19^, and mechanical sonication in organic solvents^20^ have been proposed for GNR synthesis. Among those methods, CNT longitudinal unzipping using a mixture of strong acids such as sulfuric acid (H_2_SO_4_) and oxidant(s) such as potassium permanganate (KMnO_4_) has been demonstrated an effective method to produced GNRs in a large quantity^14^. Moreover, oxygen-containing functional groups can be created on the GNRs simultaneously by this method, providing suitable GNRs with solution processability for industrial-oriented solution-based processing^21^. In this method, the critical step is to decrease the strong van der Waals attractions between the walls of CNTs by intercalating SO_4_^2−^ ions. Therefore the nanotube unzipping can start with the oxidants attaching one of the internal C-C bonds of the CNTs, stretching and then breaking it to from nanoribbon structure^14^. However usually large amount of concentrated H_2_SO_4_ (typically 1 mg/ml of CNT concentration in H_2_SO_4_) is required to unzip the nanotube completely due to the strong van der Waals interactions between the walls of CNTs^14^. From the practical point of view, a process required large amount of strong acids is not preferred for industrial setting because it is not only expensive for the waste treatment but also harmful to the environment. Therefore there is still a need to develop an environment-friendly and scalable method to produce GNRs.

The intertube intercalation of CNTs by different ions has been showed an effective method to decrease the van der Waals interactions between individual tubes and assist the exfoliation of bundled CNTs^22^,^23^,^24^. It is also possible to assist the nanotube unzipping process by intratube intercalation with suitable ions^25^. By selecting appropriate neutral molecules or ions to substitute H_2_SO_4_ as an intercalation agent shall be a promising approach to produce GNRs with a high yield and further reduce the usage of strong acid during the nanotube unzipping^14^,^24^,^25^. However, there are very few reports relevant to this issue, and a systematic study to reveal the relationship between the formation of GNRs by CNT unzipping and the molecular intercalation is still needed. Our previous work suggests that it is possible to produce GNRs by CNT unzipping with a low usage of H_2_SO_4_ and neutral molecules (e.g. KNO_3_) as an intercalation agent^4^. In this report, we perform a systematic research and describe an intercalation-assisted method to synthesis GNRs by unzipping the multi-walled CNT (MWCNT) side walls. By intertube and intratube intercalations with suitable ions under appropriate conditions, we show that it is possible to produce GNRs with a high yield using a low amount of H_2_SO_4_ (10 mg/ml of CNT concentration in H_2_SO_4_). The key step is to introduce a pretreatment of raw CNTs with potassium nitrate (KNO_3_) and H_2_SO_4_ under appropriate condition, allowing the potassium ions (K^+^), nitrate (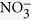) and sulfate ions (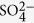) to exfoliate and debundle the bundled CNTs and further intercalate into the coaxial wall of CNTs (see Fig. 1). The next step is to use KMnO_4_ to perform CNT oxidative unzippping at appropriate conditions such as time, oxidant concentration, and temperature. Overall, it is possible to decrease the van der Waals interactions between individual nanoutbes and the coaxial walls of CNTs by this two-step method, facilitating the GNR synthesis through CNT unzipping with a low amount of H_2_SO_4_. Detailed scanning electron microscopy (SEM), transmission electron microscopy (TEM), X-ray diffraction (XRD), and micro Raman characterizations show that our method can produce GNRs with high yield. Systematic micro Raman, X-ray photoelectron spectroscopy (XPS), and XRD characterizations suggest that the KNO_3_ and H_2_SO_4_ play an important role in the intertube and intratube intercalations during the pretreatment, providing a scientific understanding how to enhance the GNR formation by accelerating the CNT unzipping with appropriate molecular intercalation.

## Result

The quality and structure of the raw CNTs for the GNR synthesis by unzipping method is critical^14^. We used MWCNTs as the starting materials because it is possible to increase the GNR yield based on the multi-shell property^26^,^27^. The MWCNTs used as the starting material in this report was synthesized through a water-assisted CVD (supporting information). SEM characterization indicates that the raw MWCNTs were with diameters and lengths ranging from 20 to 50 nm and 8 to 10 μm, respectively, and TEM image suggests that the raw MWCNTs are almost free of amorphous carbons [Fig. S1]. In addition, the low D/G ratio exhibited in the Raman spectrum [Fig. S2] shows that the raw MWCNTs are with high crystallinity, which is a good starting material for GNR synthesis^28^.

We start to discuss the effects of the intertube and intratube intercalations introduced by the pretreatment with different agents including small (sample PT1) and large (sample PT2) amounts of H_2_SO_4_, and KNO_3_ with a small amount of H_2_SO_4_ (sample PT3). The details of the sample preparation can be found in the experimental section and summarized in Table 1. We note that all the samples were prepared by the same procedure (see experimental section) so that we can study the effect of the intercalation agents on the intertube and intratube intercalations carefully. Micro Raman spectroscopy is a powerful method to study the effect of CNT debundling due to intertube intercalation^29^. Figure 2(a) shows the Raman spectra of the pristine MWCNTs and the as-treated samples. Spectra of different as-treated samples were recorded at the same Raman parameters under 532 nm excitation wavelength and were normalized with respect to the G-band intensity. The G-band corresponds to the characteristic peak of the E2g symmetry phonon mode of graphite at 1563 cm^−1^ 30,31. It is also noted that all the samples feature the D-band at 1330 cm^−1^ is due to the second-order Raman scattering process involving^31^,^32^. The D-band can be due to the structural imperfections in the carbon basal plane or edge site^33^,^34^. It is found that the G-band of pristine nanotubes (sample P) was shift from 1563 cm^−1^ to 1573 and 1576 cm^−1^ for sample PT1 and PT2, respectively [Fig. 2(a) and S3(a)]. The upshift of G-band is due to the C-C bond were shorten by the electron transfer from CNTs to the molecules (eg. SO_4_^2−^) existed in the nanotube outer-wall contacts and is the indications of the CNT debundling by intertube intercalation^29^. It can also be referred that the higher amounts of H_2_SO_4_ used in the pretreatment, the better the debundling effect. We noticed that the G-band was upshift to 1575 cm^−1^ in sample PT3 treated with KNO_3_ and a small amount of H_2_SO_4_ [Fig. 2(a) and S3(a)], which is very close to the result in sample PT2, suggesting that it is possible to have effective CNT debundling by adding KNO_3_ with a small amount of H_2_SO_4_ (10 ml) during the pretreatment.

XRD was additionally performed to understand the microstructure of pristine CNTs and as-pretreated samples, and the XRD patterns are shown in Fig. 2(b). It is found that the 2θ value of (002) graphite peak of pristine CNTs was downshifted from 26.61° to 26.42° and 26.39° in sample PT1 and PT2, respectively [Fig. 2(b) and S3(b)], indicating the interlayer distance of graphite (002), d_002_, was expanded due to the intratube intercalation. This result is in consistent with previous work^35^. We further found the 2θ value of (002) graphite peak of pristine CNTs was downshifted to 26.38° in sample PT3 treated with KNO_3_ during the pretreatment [Fig. 2(b) and S3(b)]. The increased value of d_002_ of as-pretreated samples is an indication that the sulfate and nitrate ions were co-intercalated into the coaxial graphene cylinders of MWCNTs.

To further study the intratube intercalation effect, the as-treated samples were characterized by XPS. Previous report suggests that the upshift of C1s binding energy in XPS is an indication of the intratube intercalation^35^. Figure 3 shows the C1s spectra of pristine MWCNTs and as-pretreated samples, which were carefully calibrated the C1s binding energy (BE_C1s_) at 284.5 eV and normalized with respect to the C1s peak intensity^35^. It is found that the C1s binding energy of pristine nanotubes is upshift from 284.5 eV to 285.6 and 285.2 eV in sample PT1 and PT2, respectively, indicating that the larger amount of H_2_SO_4_ used in the pretreatment, the stronger the intratube intercalation effect [Fig. 3(a–c)]. Notably, we found the C1s binding energy was also upshift from 284.5 eV to 284.9 eV in sample PT3 treated with KNO_3_ and a small amount of H_2_SO_4_ [Fig. 3(a,d)], suggesting that the improved CNT intratube intercalation by adding KNO_3_ during the pretreatment. The reason why C1s binding energy of sample PT2 is higher than sample PT3 can be explained by that the presence of K^+^ from KNO_3_^35^. The detailed C1s deconvoluted results of as-pretreated samples show several peaks including electron-poor carbon, electron-rich carbon, sp^2^ C-C, C-O, C=O, and O-C=O at, 284, 284.5, 285, 286, 287, and 289 eV^35^,^36^. Previous works suggests that the existence of electron-poor and electron-rich carbons peaks can be explained in terms of the dipolar interactions between the sp^2^ carbons and the intercalated molecules. We notice the strong peaks of electron-rich carbon regions exhibited in the XPS spectra of the as-pretreated samples, indicating the strong charge transfer between the intercalated agents (e.g. KNO_3_ and H_2_SO_4_) and the graphitic carbon of CNTs [Fig. 3(b–d)]^35^. The HRXPS result confirms that the intratube intercalation was occurred in sample PT3 treated with KNO_3_ and a small amount of H_2_SO_4_ [Fig. 3(d)]. Overall, the XPS analysis further provides the supported evidence for the observation by Raman spectra and XRD characterization, showing that the selected intercalated agent (e.g. KNO_3_ and H_2_SO_4_) can effectively intercalate into the bundle CNT structure and the coaxial walls of CNTs to decrease the strong van der Waals force of CNTs, which is critical to produce GNR with high yield by unzipping CNTs (discuss below).

**Figure 1:**
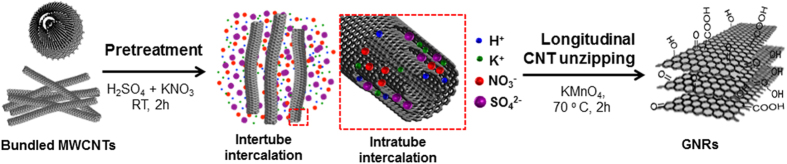
Illustration of a two-step oxidative process to synthesis GNRs by MWCNT longitudinal unzipping. The first step is to use a pretreatment of raw MWCNTs with KNO_3_ and H_2_SO_4_ under appropriate conditions, allowing the K^+^, 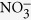, and 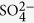 ions to exfoliate the bundled CNTs and intercalate into the coaxial walls of CNTs by intertube and intratube intercalation, respectively. The second step is to use the KMnO_4_ as the oxidant to longitudinally unzip MWCNTs under suitable condition.

**Figure 2:**
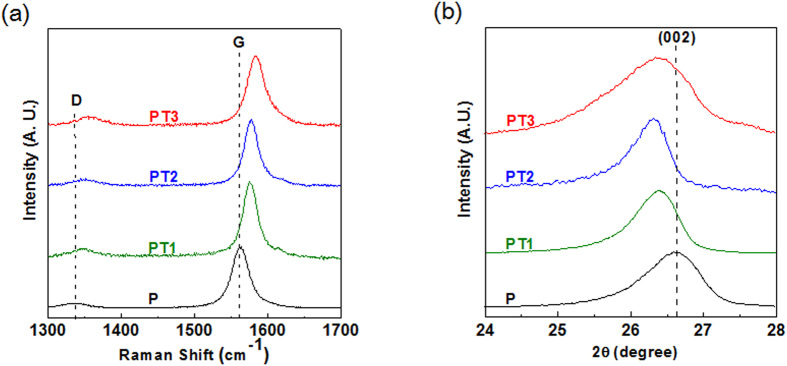
(**a**) Micro Raman spectra of pristine CNTs and different as-pretreated samples. The spectra were normalized by the sample G-band intensity and offset for clarity.(**b**) XRD patterns of pristine CNTs and different as-pretreated samples. The patterns were normalized by the sample intensity at (002) diffraction angel and offset for clarity. Pristine CNTs are denoted as P. (All reaction condition are detailed in Table 1).

**Figure 3:**
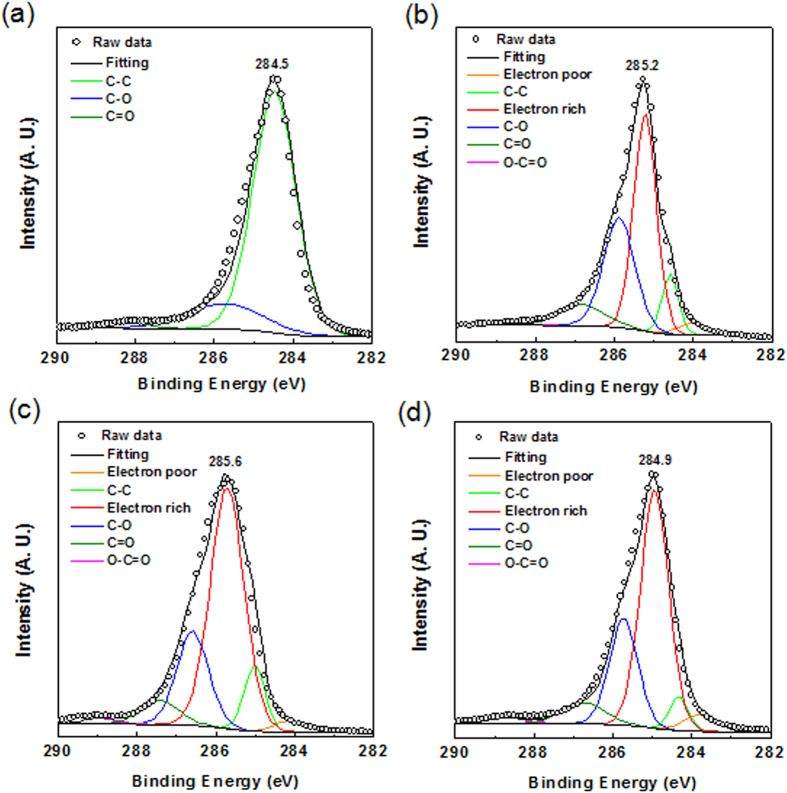
HRXPS C1s spectra of (**a**) pristine CNTs and different as-pretreated samples : (**b**) PT1, (**c**) PT2, and (**d**) PT3. (All reaction condition are detailed in Table 1).

**Figure 4:**
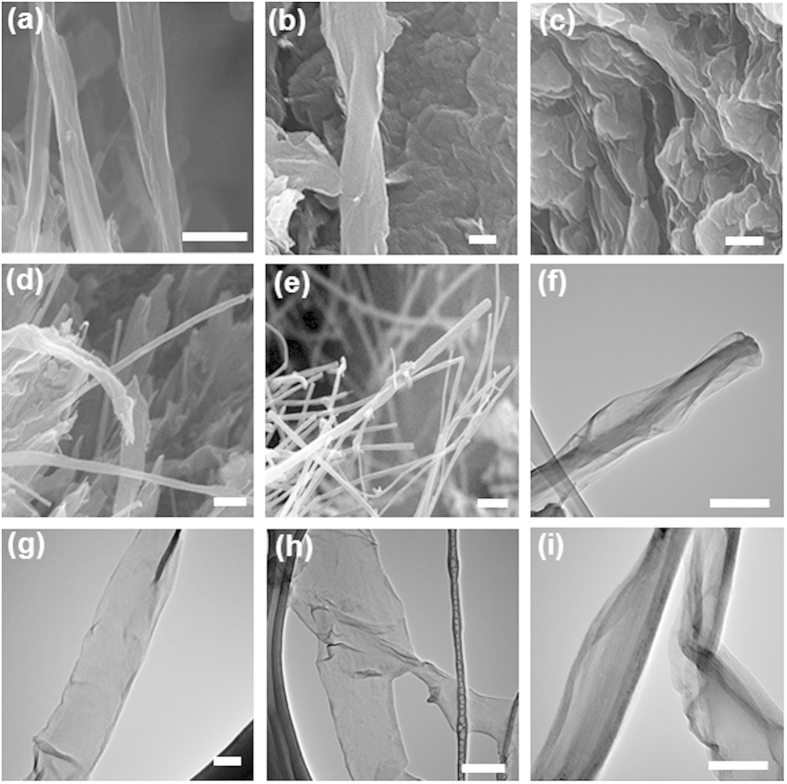
SEM images of (**a**) sample A, (**b**) sample B, (**c**) sample C, (**d**) sample D, and (**e**) sample E. (Scale bar = 50 nm). TEM images of (**f**) sample A, (**g**) sample B, (**h**) sample C, and (**i**) sample D. (Scale bar = 50 nm). All the samples were synthesized using the developed stepwise, solution-based oxidative process shown in Fig. 1. Reaction condition are detailed in Table 2.

**Figure 5:**
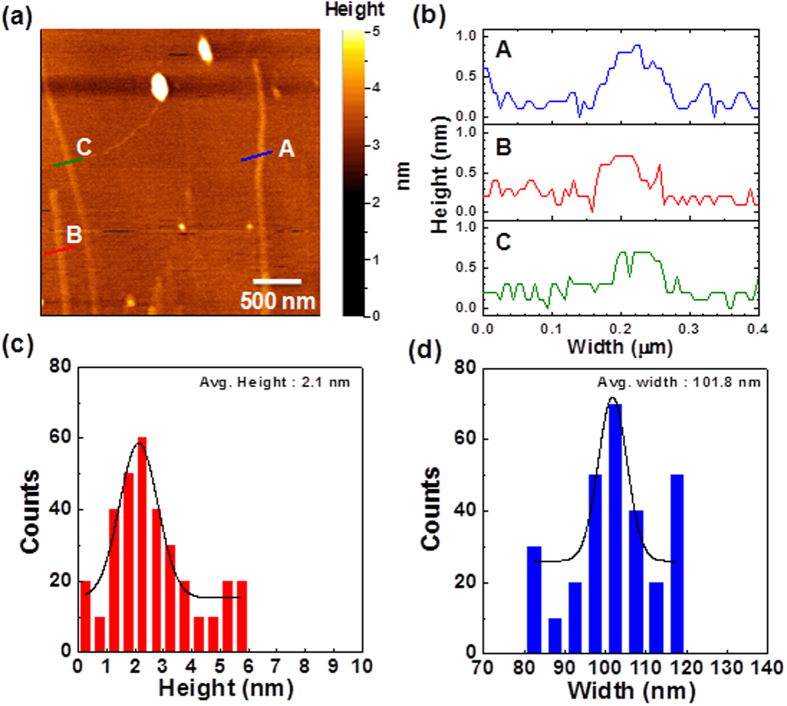
(**a**) AFM image of Sample B. Reaction condition is detailed in Table 2. (**b**) The height data of three individual GNRs shown in (**a**). The A, B, and C are used to denote the individual GNRs. (**c**) Height and (**d**) width distributions measured by AFM. Solid lines represent the Gaussian distributions to the experimental data.

After the pretreatment, all the as-treated CNTs were reacted with the same amount of KMnO_4_ as the oxidant to unzip the as-treated CNTs. To understand the effectiveness and yield of the GNR production using the developed pretreatment with different agents and different unzipping conditions, a series of characterizations including SEM, TEM, atomic force microscopy (AFM), XRD, micro Raman, XPS, Fourier-transform infrared spectroscopy (FTIR), and thermogravimetric (TGA) were performed. Detailed reaction conditions of different as-produced samples are summarized in the Table 2. Representative SEM and TEM images are shown in Fig. 4. Below the temperature of 30 °C, we did not observe any longitudinal CNT unzipping by 10 ml H_2_SO_4_ with KNO_3_ under the extensive microscopy characterization. As the reaction temperature was increased to 30 °C, it is found that the raw MWCNTs [Fig. S1] were partially unzipped from the outer layer of cylindrical graphene sheets by 10 ml H_2_SO_4_ with KNO_3_ (sample A), according to the SEM observation [Fig. 4(a)]. Additional TEM characterization indicates the incompletely unzipped CNTs with core shell structure inside the unzipped ribbons [Fig. 4(f)], suggesting that 30 °C might be the onset temperature of CNT longitudinal unzipping in our work, which is in agreed with the observation of previous work^36^. As the temperature was further increased to 70 °C (sample B), it was found that the local part of raw CNTs were formed fully unzipped ribbon structures [Fig. 4(b)]. The low magnification TEM images were also shown in Fig. S4(a), revealing the unzipped ribbons with irregular winkles in the surface and the edge. The result were further support by the high magnification TEM observation [Fig. 4(g)] that the structure of sample B were strip of ribbon with width near 180 nm, which is about 3 times larger than the average diameter of pristine CNTs [Fig. S1]. We note that it is difficult to image the edge structures of the as-produced samples by TEM due to the edge oxidation and curling. While it is possible to remove the oxygen-containing groups by heating the samples over 2000 °C, the reconstruction of the as-produce GNRs will be occurred during the high temperature treatment. The overall morphology and physical structure of the sample B was further carefully investigated using AFM. Figure 5(a) shows the representative AFM image and indicates the presence of single atomic layer ribbon nanostructures [Fig. 5(b)] with high edge linearity. The histogram and Gaussian statistical analysis shown in Fig. 5(c) revealed the single-layer to few-layer nanostructures indicated by the averaged nominal height of 2.1 nm. In addition, the nominal widths of the as-produced GNRs were ranging from 80 to 120 nm and an averaged nominal width of 101.8 nm [Fig. 5(d)]. The AFM result is in agreed with the TEM observation, confirming that single-layered GNRs with high edge linearity can be produced by our develop method (Fig. 1).

We notice that the morphology of sample B produced with KNO_3_ and 10 ml H_2_SO_4_ at 70 °C is very similar to that of the GNRs produced with 100 ml H_2_SO_4_ at 70 °C (sample C) [Fig. 4(c,h)]. To further understand the role of KNO_3_ on the CNT longitudinal unzipping, two samples treated with 10 ml H_2_SO_4_ (sample D) and 10 ml KNO_3(aq)_ (sample E) respectively were prepared and characterized by SEM and TEM. The images show that a mixture of CNTs and partially unzipped CNTs and solely tubular microstructure were existed in sample D [Fig. 4(d,i), and S4(b)] and sample E [Fig. 4(e)], respectively. The result suggests that first of all, H_2_SO_4_ is essential to produce GNR by CNT unzipping, which is consistent with previous study that the CNT opening is based on the oxidation of alkenes by permanganate in strong acid^14^. Secondary, the formation of GNRs by CNT unzipping could not be completed if the amount of H_2_SO_4_ used in the entire reaction was too low [Fig. 4(d,i), and S3(b)]. By adding KNO_3_ to assist the intertube and intratube intercalations of CNTs during the preferment step, it is possible to reduce the usage of H_2_SO_4_for GNR production by CNT unzipping [Fig. 4(b,g), and S3(a)].

**Figure 6:**
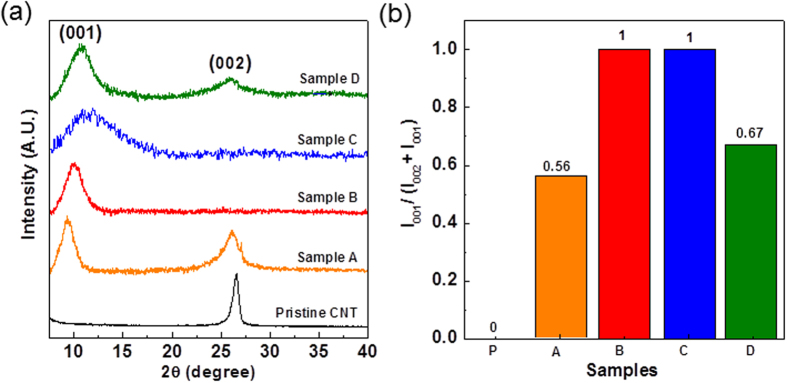
(**a**) XRD patterns of pristine CNTs and different as-pretreated samples. Reaction condition are detailed in Table 2. (002) and (001) are denoted as the planes of the interplanar graphite of CNTs and oxidized GNRs, respectively. (**b**) Quantitative analysis of GNR yield by the ratio of I_001_**/**(I_002_ + I_001_) in the XRD patterns^35^,^38^. (I_001_) and (I_002_) indicate the peak intensities of oxidized GNR and CNT, respectively.

The additional evidence of the microstructure changes of the starting CNTs and the as-produced samples were obtained from the XRD characterization. Moreover, the XRD analysis can be performed to quantitatively determine the degree of unzipping^38^. In the XRD patterns, the raw MWCNTs shows a peak at 26.6° [Fig. 6(a)], attributed to the (002) plane of the interplanar graphite with a d spacing of 0.34 nm according to the Bragg’s Law^37^. For the sample A prepared at 30 °C [Fig. 6(a)], a characteristic peak of oxidized GNRs structure at 9.6° was shown^14^,^38^, suggesting the existence of a mixture of MWCNTs and oxidized GNRs. This result is in consistent with SEM observation [Fig. 4(b)] and previous work^14^,^38^. The calculated d spacing was increased from 0.34 to 0.82 nm, which was due to the functional groups generated between the adjacent layers of as-produced GNRs during the process of oxidative unzipping CNTs^39^. As the temperature was increased to 70 °C (sample B), the XRD pattern shows no characteristic (002) peak of CNTs and only oxidized GNR peak at 10.0°, implying that a high portion of oxidized GNRs was existed in this as-produced sample. Furthermore, it is found both two peaks at 10.0° and 26.6° in the XRD pattern of sample D, indicating that sample D was composed with both oxidized GNR and CNT. Further quantitative analysis of oxidized GNR portion can be evaluated by the ratio of I_001_**/**(I_002_ + I_001_) in the XRD patterns^35^,^37^. The (I_001_) and (I_002_) indicate the peak intensities of oxidized GNR and CNT, respectively. Fig. 6(b) shows the estimated result of I_001_**/**(I_002_ + I_001_) ratios of various samples. We notice that the ratio of sample B treated with KNO_3_ and 10 ml H_2_SO_4_ is nearly 1, suggesting a nearly 100% yield of oxidized GNR. In addition the ratio of sample B is 1.49 times higher than that of sample D treat with 10 ml H_2_SO_4_ (0.67), indicating that GNRs with high yield could be generated in the report method using KNO_3_ to assist the CNT unzipping.

**Figure 7:**
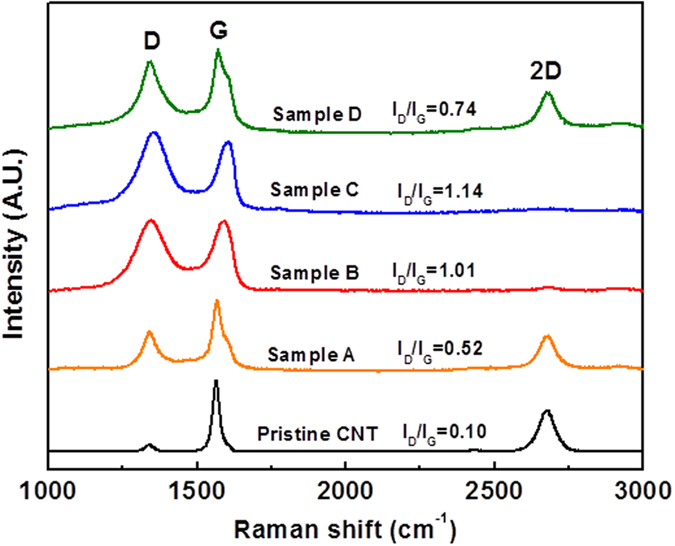
Micro Raman spectra of pristine CNTs and as-produced samples. Reaction conditions are detailed in Table 2. The spectra were normalized by the sample G-band intensity and offset for clarity.

Further atomic-scale structural information of the as-produced GNRs can be obtained from micro Raman spectroscopy characterization. Figure 7 shows the representative Raman spectra of pristine CNTs and different as-produced GNRs. Three bands are present around 1340 cm^−1^, 1590 cm^−1^, and 2675 cm^−1^, which are respectively assigned to the D-band, G-band, and 2D-band of carbon. The D-band can be due to the structural imperfections in the carbon basal plane or edge site^33^,^34^ while the G-band is the representative peak of graphite, corresponding to the sp^2^ carbon bond stretching of E2g mode^33^. The 2D-band is the overtone of the D-band and typically used to indicate the quality of graphene structure^31^,^40^. After the oxidative unzipping process, the intensity of D-and 2D-band increased and decreased, respectively, indicating that the-produced GNRs contained more defects or edge effect than starting CNTs. The defects can be holes with different sizes and shapes on the basal planes of the as-produced GNRs and the attached oxygen functional groups at the edges and surface of ribbons^36^. The defects were caused by the unzipping process including manganate ester formation and cleavage of C-C bonding introduced by the vicinal diols^36^. The Raman analysis further supports the XRD results [Fig. 6a]. Generally the value of the intensity ratio of D-band to G-band (I_D_/I_G_) is used to estimate the degree of disorder and defects of carbon materials^41^. The I_D_/I_G_ ratio of sample B and C were 1.01 and 1.14, respectively, showing that more defect generated on the as-produced GNR by large amount of H_2_SO_4_ (100 ml)^36^. We further noticed that the I_D_/I_G_ ratio of sample B and sample D were 1.01 and 0.74 respectively, indicating that sample B was with higher level of oxidation than sample D while both samples were synthesized by a low amount of H_2_SO_4_ (10 ml). This finding suggests that it is possible to achieve higher level of oxidation with the assistance of KNO_3_ during the nanotube unzipping.

**Figure 8:**
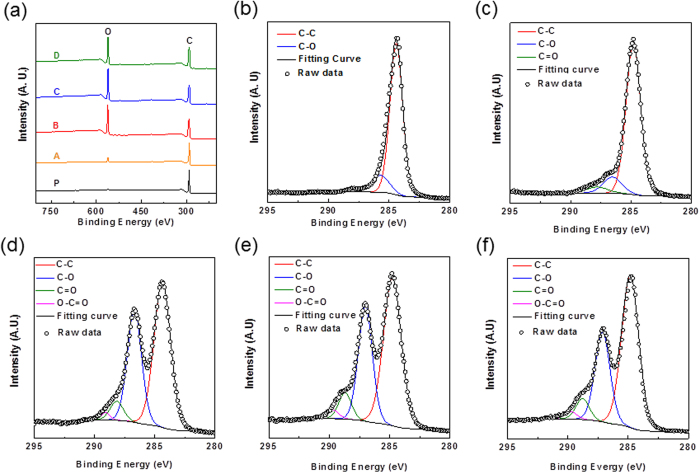
(**a**) XPS spectra of pristine CNTs and different as-synthesized samples.Reaction condition are detailed in Table 2. The labels of C and O are indicated as the C1s and O1s peaks respectively at. 284.6 eV and 534 eV, respectively. HRXPS C1s spectra of (**b**) pristine CNTs, (**c**) sample A, (**d**) sample B, (**e**) sample C, and (**f**) sample D. The C1s peaks were carefully deconvoluted to C-C, C-O, C=O, and COOH surface functionalities at 284.4, 286, 287, and 289 eV, respectively.

The chemical compositions of as-produced samples were systematically studied by XPS. Figure 8(a) shows the XPS survey scan spectra of pristine CNTs and as-produced GNRs and were normalized with respect to the C1s intensity. It is noted that only C1s and O1s peaks respectively at 284.5 eV and 534 eV were observed in the XPS spectra of as-produced GNRs, showing no impurities such as the moieties from KNO_3_ and H_2_SO_4_ in the as-produced samples. In general, the ratio of peak intensity of C1s to that of O1s (C/O ratio) in the XPS spectra of carbon materials is an indication of the oxidation degree of carbon materials^41^,^42^. The C/O ratios of different samples were summarized in Table 3. It is found that the C/O ratios were largely decreased from 19 to 2.5 for samples prepared at 30 °C and 70 °C, respectively, suggesting that temperature is a critical factor to control the oxidation degree of as-produced GNRs. Moreover, we found that the C/O ratio of sample B is similar to those of sample C and D, suggesting the oxidation degree of the as-produced GNRs prepared by the mixture of KNO_3_ and H_2_SO_4_ is similar to those of samples prepared with H_2_SO_4_, which is in consistent with the Raman result. The surface functionalities of the as-produced samples were further studied by HRXPS. Figure 8(b–f) show the C1s peaks of HRXPS spectra of pristine CNTs and as-produced GNRs and can be deconvoluted to several peaks at 284.4, 286, 287, and 289 eV, which are corresponded to sp^2^ C-C, C-O, C=O, and COOH surface functionalities, respectively^36^. This result suggests that the as-produced GNRs possess oxygen-containing functional groups, which is in agreed with our XRD and Raman results as well as previous study^14^,^36^,^43^. The quantitative fractions of the oxygen-containing functional groups in the as-produced GNRs are summarized in Table 3. The result shows that sample B prepared with the mixture of KNO_3_ and 10 ml H_2_SO_4_ with higher fraction of surface functionality of 42.91 at% [Fig. 8(d)] than that of sample D reacted with 10 ml H_2_SO_4_ (39.40 at%) [Fig. 8(f)], implying that the degree of surface functionalization of GNRs during the chemical oxidative unzipping CNTs can be enhanced by introducing KNO_3_ in the pretreatment. This finding is also confirmed the I_D_/I_G_ ratio analysis in our micro Raman characterization [Fig. 7]. While sample C prepared with 100 ml H_2_SO_4_ shows a higher surface functionality [Fig. 8(e)] than that of sample B prepared by the mixture of KNO_3_ and 10 ml H_2_SO_4_ (Table 3), a higher COOH (2.93%) shown in the C1s region indicates more defects generated due to the large amount of sulfuric acid used in the sample preparation^36^.

**Figure 9:**
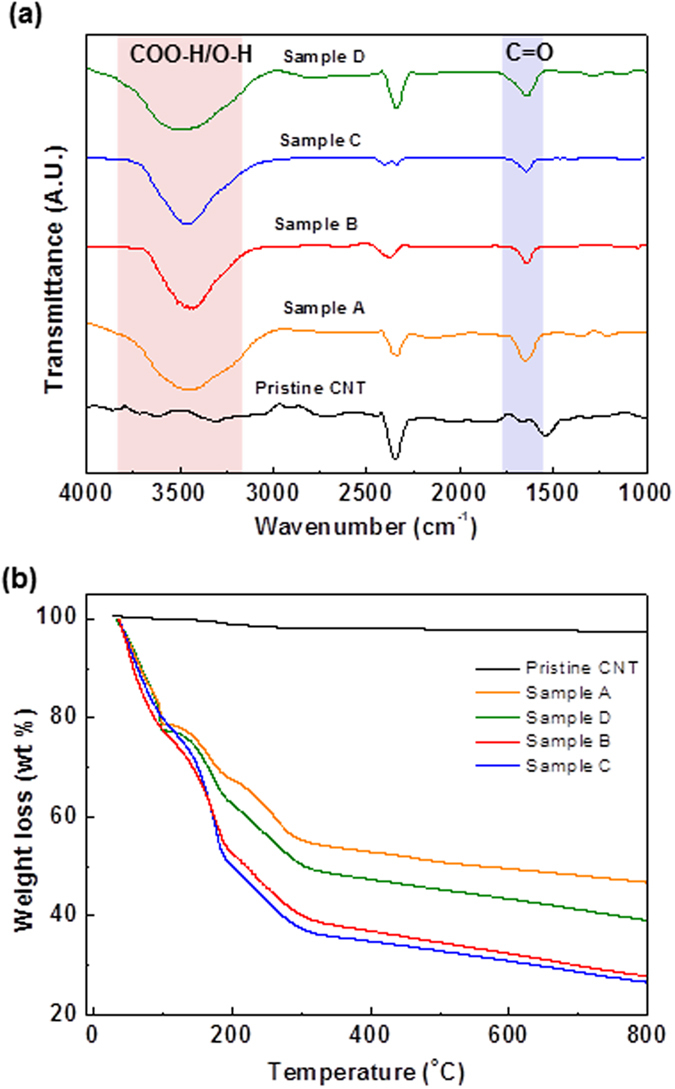
(**a**) FTIR spectra of pristine CNTs and different as-synthesized samples. Reaction condition are detailed in Table 2. The labels of C=O and COO-H/O-H are indicated as C=O and COO-H/O-H stretching modes, respectively at 1647 and 3445 cm^−1^, respectively. (**b**) TGA patterns of pristine CNTs and different as-synthesized samples.

FTIR and TGA were also performed to verify the Raman and XPS results. The FTIR spectrum of the pristine nanotubes is practically featureless with extremely low infrared absorption intensities [Fig. 9(a)]. Two major peaks at 1647 cm^−1^ (C-O stretching) and 3445 cm^−1^ (COO-H/O-H stretching) exhibited for the as-produced samples suggest that various carboxyl and hydroxyl functionalities were existed on the as-produced GNRs. The result were consisted with the XPS study [Fig. 8], which can be attributed to the formation of epoxy, carbonyl, and carboxyl groups generated during the CNTs oxidative unzipping process^14^,^36^,^43^. To provide further supported evidence for the observation of XPS and FTIR, TGA was performed to study the total weight loss (wt%) of as-produced samples [Fig. 9(b)]. The weight loss curve of pristine CNTs showed a slight drop after 600 °C, which can be attributed to the residual impurities or catalysts (i.e. iron) during the CNTs synthesis^44^. For weight losses of the as-produced GNRs between 150 and 300 °C were corresponded to the formation of CO, CO_2_ gas release from the labile oxygen-containing surface functionalities of the ribbons^45^. For 350 to 800 °C, the weight losses indicated the remove of other more stable oxygen-containing functional group^46^. The weight loss (wt%) were increased in an order: sample P > A > D > B > C. The results imply that the level of oxidation for all samples, and can be further confirmed the result the C/O ratio by XPS of as-produced samples (Table 3).

## Discussion

To date, the mechanism of GNR synthesis by longitudinal unzipping of CNTs is still not clear, making it difficult to predict the optimal condition to produce GNRs with high yield. On the basis of the experimental result (Table S1), we propose the possible mechanism in our system discussed in the following. First of all, sulfate ions can enter the interspace between CNTs through oxidation, leading to the intertube interaction to reduce the inter-CNT van der Waals force (Sample C and D)^47^,^48^. In addition, the nitrate ions can act as the co-intercalatent to assist the intertube interaction (Sample B). Furthermore K^+^ ions can enter into the graphene layers of CNTs if the interaction energy overcomes the net energy of inter-CNT van der Waals interaction (Sample A and B)^49^. After the interaction assisted by sulfate and nitrate ions and K^+^ ions, the reactive MnO^4−^ with H_2_SO_4_ can enter the expanded interspace of CNTs, and then oxidize and unzip the C=C bonds. The unzipping of C=C bond on the outer layer of MWCNTs was triggered by sufficient number of defects created by mild oxidizer condition at edge sites and grain boundaries of the CNTs. The defective sites at the edges or grain boundaries open up due to intercalation by K^+^ ions and solvated sulfate and nitrate ions. The proposed mechanism can be supported by the previous work which suggests that the sulfate and nitrate ions can act as intercalatent and co-intercalatent, respectively, along with K^+^ ions, to cause exfoliation of MWCNTs in an effective manner^25^. To understand the role of K^+^ ions during the intercalating process, we have performed control experiments using various potassium salts including KNO_3_, K_2_CO_3_, and K_2_S_2_O_8_ with different concentrations in the pretreatment step to unzip the CNTs. We found that KNO_3_ in H_2_SO_4_ (sample B, F, and G) were more effective for exfoliating MWCNTs compared to K_2_CO_3_ (Sample H, I, and J) and K_2_S_2_O_8_ (Sample K, L, and M) in H_2_SO_4_. The exfoliation efficiency of CNTs in 2 M KNO_3_ (sample G) was lower than that in 1 M KNO_3_ (sample B), and the corresponding yield of GNRs is 80% and 100%, respectively. This may be the reason that with higher concentration of KNO_3_, more energy is required to initiate intercalation between two graphene layers of CNTs. The low exfoliation efficiency of CNTs in diluted KNO_3_ (0.5 M; sample F) is most likely due to the inefficient intercalation of anions and K^+^ ions^25^. The above results suggest that K^+^ ions may play an important role in the interspace CNT intercalation. In general the chemical affinities and the sizes and bonding distances of the intercalatent can affect the intercalation in MWCNTs^50^. However, the exact mechanism is still not clear due to the intricacies associated with modulating the electrostatic, solvation, and van der Waals interaction by changing ionic strength, conductivity, solvation energy, viscosity, etc. Nevertheless, our work provides the experimental understanding of the intercalation effect on the CNT unzipping. Further theoretical and experimental studies devoted to the rational design of different intercalation molecules will be helpful to provide a fundamental understanding and further enhance the production yield of GNRs with lower strong acid usage by CNT longitudinal unzipping.

## Conclusion

In conclusion, we report a facile and effective solution-based oxidative process to produce GNRs by CNT longitudinal unzipping. The experimental finding presented in this report shows that the intertube and intratube intercalation of CNTs by suitable intercalation molecules can be a useful method to achieve not only high-yield GNR synthesis but also low usage of concentrated H_2_SO_4_. The experimental approach should allow other materials synthesis or processing related to the intercalation of layered materials including graphene, boron nitride, and transition metal dichalcogenides nanosheets^51^,^52^.

## Methods

Potassium permanganate (KMnO_4_, 98%), hydrogen peroxide (H_2_O_2_, 35%), and ether [(C_2_H_5_)_2_O, 99+%] were obtained from ACROS. Potassium nitrate (KNO_3_, 95%), potassium carbonate (K_2_CO_3_, 99%), and potassium persulfate (K_2_S_2_O_8_, 99%) were purchased from JT-Baker. Hydrochloric acid (HCl, 37%) and sulfuric acid (H_2_SO_4_, >95%) were purchased from Scharlau. All the chemicals were used as received. The raw MWCNTs were synthesized using a water-assisted catalytic chemical vapor deposition^53^, and details are described in the supporting information. The detailed GNR synthesis process was similar to our previous work^4^ and provided in the supporting information. In brief, MWCNTs (0.1 g) was magnetically stirred (300 rpm, 2 hrs) with a different amount of concentrated H_2_SO_4_ and 1 g KNO_3._ Detailed conditions are summarized in Table 1. Then 0.5 g KMnO_4_ was added slowly to the solution and further stirred (2 hrs, room temperature). Then the unzipping reaction was performed for 2 hrs in a water bath. Table 2 shows the detailed reaction conditions. After the reaction was completed, the product was purified and dried with a series steps reported elsewhere^14^. The as-produced samples were characterized by various techniques including SEM, TEM, AFM, XPS, XRD, micro Raman, FTIR, and TGA. The detailed sample preparations and measurement conditions are provided in the supporting information.

## Additional Information

**How to cite this article**: Li, Y.-S. *et al.* Intercalation-assisted longitudinal unzipping of carbon nanotubes for green and scalable synthesis of graphene nanoribbons. *Sci. Rep.* **6**, 22755; doi: 10.1038/srep22755 (2016).

## Supplementary Material

Supplementary Information

## Figures and Tables

**Table 1:** The summarized reaction conditions of MWCNT pretreatment.

Sample	H_2_SO_4_ (ml)	KNO_3_ (M)
P	–	–
PT1	10	0
PT2	100	0
PT3	10	1

P denotes the pristine MWCNT. The amount of raw MWCNT is 0.1 g.

**Table 2:** The summarized reaction conditions of MWCNT longitudinal unzipping.

Sample	H_2_SO_4_ (ml)	KNO_3_ (M)	Temperature (°C)
A	10	1	30
B	10	1	70
C	100	0	70
D	10	0	70
E	0	1^a^	70

The amount of raw MWCNT is 0.1 g.

^a^Dissolve in 10 ml DI water.

**Table 3:** Atomic percentages of different carbon bonds indentified by XPS in the pristine CNTs and different as-produced GNRs.

Sample	C/O	C1s (at%)	C-O (at%)	C=O (at%)	COOH (at%)
P	24.00	89.97	10.03	0	0
A	19.00	83.24	11.57	5.19	0
B	2.50	56.94	35.17	6.28	1.61
C	2.20	55.21	34.72	7.14	2.93
D	2.90	60.60	30.93	6.46	2.01

## References

[b1] Li X., Wang X., Zhang L., Lee S. & Dai H. Chemically Derived, Ultrasmooth Graphene Nanoribbon Semiconductors. Science 319, 1229–1232 (2008). 18218865 10.1126/science.1150878

[b2] Son Y.-W., Cohen M. L. & Louie S. G. Half-metallic graphene nanoribbons. Nature 444, 347–349 (2006). 17108960 10.1038/nature05180

[b3] Han M. Y., Özyilmaz B., Zhang Y. & Kim P. Energy band-gap engineering of graphene nanoribbons. Phys. Rev. lett. 98, 206805 (2007). 17677729 10.1103/PhysRevLett.98.206805

[b4] Wang C., Li Y.-S., Jiang J. & Chiang W.-H. Controllable Tailoring Graphene Nanoribbons with Tunable Surface Functionalities: An Effective Strategy toward High-Performance Lithium-Ion Batteries. ACS Appl. Mater. Interfaces 7, 17441–17449 (2015). 26196904 10.1021/acsami.5b04864

[b5] Wang Y., Shao Y., Matson D. W., Li J. & Lin Y. Nitrogen-doped graphene and its application in electrochemical biosensing. ACS Nano 4, 1790–1798 (2010). 20373745 10.1021/nn100315s

[b6] Liu Y., Dong X. & Chen P. Biological and chemical sensors based on graphene materials. Chem. So. Rev. 41, 2283–2307 (2012). 10.1039/c1cs15270j 22143223

[b7] Holby E. F. & Taylor C. D. Control of graphene nanoribbon vacancies by Fe and N dopants: Implications for catalysis. Appl. Phys. Lett. 101, 064102 (2012).

[b8] Davis D. J. *et al.* Silver‐Graphene Nanoribbon Composite Catalyst for the Oxygen Reduction Reaction in Alkaline Electrolyte. Electroanal. 26, 164–170 (2014).

[b9] Kim H., Abdala A. A. & Macosko C. W. Graphene/polymer nanocomposites. Macromolecules 43, 6515–6530 (2010).

[b10] Lin J. *et al.* Graphene nanoribbon and nanostructured SnO2 composite anodes for lithium ion batteries. ACS Nano 7, 6001–6006 (2013). 23758123 10.1021/nn4016899

[b11] Wang X. *et al.* Room-Temperature All-Semiconducting Sub-10-nm Graphene Nanoribbon Field-Effect Transistors. Phys. Rev. Lett. 100, 206803 (2008). 18518566 10.1103/PhysRevLett.100.206803

[b12] Fiori G. & Iannaccone G. Simulation of graphene nanoribbon field-effect transistors. Electron Device Lett., IEEE 28, 760–762 (2007).

[b13] Ma L., Wang J. & Ding F. Recent Progress and Challenges in Graphene Nanoribbon Synthesis. Chem. Phys. Chem. 14, 47–54 (2013). 22615215 10.1002/cphc.201200253

[b14] Kosynkin D. V. *et al.* Longitudinal unzipping of carbon nanotubes to form graphene nanoribbons. Nature 458, 872–876 (2009). 19370030 10.1038/nature07872

[b15] Jiao L., Zhang L., Wang X., Diankov G. & Dai H. Narrow graphene nanoribbons from carbon nanotubes. Nature 458, 877–880 (2009). 19370031 10.1038/nature07919

[b16] Elías A. L. *et al.* Longitudinal Cutting of Pure and Doped Carbon Nanotubes to Form Graphitic Nanoribbons Using Metal Clusters as Nanoscalpels. Nano Lett. 10, 366–372 (2010). 19691280 10.1021/nl901631z

[b17] Parashar U. K., Bhandari S., Srivastava R. K., Jariwala D. & Srivastava A. Single step synthesis of graphene nanoribbons by catalyst particle size dependent cutting of multiwalled carbon nanotubes. Nanoscale 3, 3876–3882 (2011). 21842103 10.1039/c1nr10483g

[b18] Cano-Márquez A. G. *et al.* Ex-MWNTs: Graphene Sheets and Ribbons Produced by Lithium Intercalation and Exfoliation of Carbon Nanotubes. Nano Lett. 9, 1527–1533 (2009). 19260705 10.1021/nl803585s

[b19] Xie L. *et al.* Graphene Nanoribbons from Unzipped Carbon Nanotubes: Atomic Structures, Raman Spectroscopy, and Electrical Properties. J. Am. Chem. Soc. 133, 10394–10397 (2011). 21678963 10.1021/ja203860a

[b20] Jiao L., Wang X., Diankov G., Wang H. & Dai H. Facile synthesis of high-quality graphene nanoribbons. Nat Nanotechnol. 5, 321–325 (2010). 20364133 10.1038/nnano.2010.54

[b21] Li D., Muller M. B., Gilje S., Kaner R. B. & Wallace G. G. Processable aqueous dispersions of graphene nanosheets. Nat Nanotechnol. 3, 101–105 (2008). 18654470 10.1038/nnano.2007.451

[b22] Bower C., Kleinhammes A., Wu Y. & Zhou O. Intercalation and partial exfoliation of single-walled carbon nanotubes by nitric acid. Chem. Phys. Lett. 288, 481–486 (1998).

[b23] Lee R. S., Kim H. J., Fischer J. E., Thess A. & Smalley R. E. Conductivity enhancement in single-walled carbon nanotube bundles doped with K and Br. Nature 388, 255–257 (1997).

[b24] Ericson L. M. *et al.* Macroscopic, Neat, Single-Walled Carbon Nanotube Fibers. Science 305, 1447–1450 (2004). 15353797 10.1126/science.1101398

[b25] Shinde D. B., Majumder M. & Pillai V. K. Counter-ion dependent, longitudinal unzipping of multi-walled carbon nanotubes to highly conductive and transparent graphene nanoribbons. Sci. Rep. 4, 4363; 10.1038/srep04363 (2014). 24621526 PMC3952148

[b26] Lin L.-Y. *et al.* A novel core-shell multi-walled carbon nanotube@graphene oxide nanoribbon heterostructure as a potential supercapacitor material. J. Mater. Chem. A 1, 11237–11245 (2013).

[b27] Wang H., Wang Y., Hu Z. & Wang X. Cutting and unzipping multiwalled carbon nanotubes into curved graphene nanosheets and their enhanced supercapacitor performance. ACS Appl. Mater. Interfaces 4, 6827–6834 (2012). 23148646 10.1021/am302000z

[b28] Dresselhaus M. S., Dresselhaus G., Saito R. & Jorio A. Raman spectroscopy of carbon nanotubes. Phys. Rep. 409, 47–99 (2005).

[b29] Rao A. M., Eklund P. C., Bandow S., Thess A. & Smalley R. E. Evidence for charge transfer in doped carbon nanotube bundles from Raman scattering. Nature 388, 257–259 (1997).

[b30] Tuinstra F. & Koenig J. L. Raman spectrum of graphite. J. Chem. Phys. 53, 1126–1130 (1970).

[b31] Ferrari A. C. *et al.* Raman spectrum of graphene and graphene layers. Phys. Rev. Lett. 97, 187401 (2006). 17155573 10.1103/PhysRevLett.97.187401

[b32] Nemanich R. J. & Solin S. A. First-and second-order Raman scattering from finite-size crystals of graphite. Phys. Rev. B 20, 392 (1979).

[b33] Malard L. M., Pimenta M. A., Dresselhaus G. & Dresselhaus M. S. Raman spectroscopy in graphene. Phys. Rep. 473, 51–87 (2009).

[b34] Ferrari A. C. & Robertson J. Interpretation of Raman spectra of disordered and amorphous carbon. Phys. Rev. B 61, 14095 (2000).

[b35] Kovtyukhova N. I. *et al.* Non-oxidative intercalation and exfoliation of graphite by Brønsted acids. Nat. Chem. 6, 957–963 (2014). 25343599 10.1038/nchem.2054

[b36] Higginbotham A. L., Kosynkin D. V., Sinitskii A., Sun Z. & Tour J. M. Lower-Defect Graphene Oxide Nanoribbons from Multiwalled Carbon Nanotubes. ACS Nano 4, 2059–2069 (2010). 20201538 10.1021/nn100118m

[b37] Yang Z. *et al.* Carbon nanotubes bridged with graphene nanoribbons and their use in high‐efficiency dye‐sensitized solar cells. Angew. Chem. Int. Ed. 52, 3996–3999 (2013). 10.1002/anie.201209736 23401014

[b38] Futaba D. N., Yamada T., Kobashi K., Yumura M. & Hata K. Macroscopic wall number analysis of single-walled, double-walled, and few-walled carbon nanotubes by X-ray diffraction. J. Am. Chem. Soc. 133, 5716–5719 (2011). 21438641 10.1021/ja2005994

[b39] Hummers W. S. & Offeman R. E. Preparation of Graphitic Oxide. J. Am. Chem. Soc. 80, 1339–1339 (1958).

[b40] Graf D. *et al.* Spatially resolved Raman spectroscopy of single-and few-layer graphene. Nano Lett. 7, 238–242 (2007). 17297984 10.1021/nl061702a

[b41] Dreyer D. R., Park S., Bielawski C. W. & Ruoff R. S. The chemistry of graphene oxide. Chem. Soc. Rev. 39, 228–240 (2010). 20023850 10.1039/b917103g

[b42] Yang D. *et al.* Chemical analysis of graphene oxide films after heat and chemical treatments by X-ray photoelectron and Micro-Raman spectroscopy. Carbon 47, 145–152 (2009).

[b43] Cataldo F. *et al.* Graphene nanoribbons produced by the oxidative unzipping of single-wall carbon nanotubes. Carbon 48, 2596–2602 (2010).

[b44] Bom D. *et al.* Thermogravimetric analysis of the oxidation of multiwalled carbon nanotubes: evidence for the role of defect sites in carbon nanotube chemistry. Nano Lett. 2, 615–619 (2002).

[b45] Stankovich S. *et al.* Synthesis of graphene-based nanosheets via chemical reduction of exfoliated graphite oxide. Carbon 45, 1558–1565 (2007).

[b46] Shen J. *et al.* Fast and facile preparation of graphene oxide and reduced graphene oxide nanoplatelets. Chem. Mater. 21, 3514–3520 (2009).

[b47] Toyoda M., Kaburagi Y., Yoshida A. & Inagaki M. Acceleration of graphitization in carbon fibers through exfoliation. Carbon 42, 2567–2572 (2004).

[b48] Inagaki M., Tashiro R., Washino Y.-i. & Toyoda M. Exfoliation process of graphite via intercalation compounds with sulfuric acid. J. Phys. Chem. Solids 65, 133–137 (2004).

[b49] Suzuki S. & Tomita M. Observation of potassium‐intercalated carbon nanotubes and their valence‐band excitation spectra. J. Appl. Phys. 79, 3739–3743 (1996).

[b50] Dresselhaus M. S. & Dresselhaus G. Intercalation compounds of graphite. Adv. Phys. 51, 1–186 (2002).

[b51] Halim U. *et al.* A rational design of cosolvent exfoliation of layered materials by directly probing liquid–solid interaction. Nat. Commun. 4, 2213; 10.1038/ncomms3213 (2013). 23896793 PMC4249658

[b52] Coleman J. N. *et al.* Two-dimensional nanosheets produced by liquid exfoliation of layered materials. Science 331, 568–571 (2011). 21292974 10.1126/science.1194975

[b53] Chiang W.-H., Futaba D. N., Yumura M. & Hata K. Growth control of single-walled, double-walled, and triple-walled carbon nanotube forests by a priori electrical resistance measurement of catalyst films. Carbon 49, 4368–4375 (2011).

