# Supplementary material for: Intercalation-assisted longitudinal unzipping of carbon nanotubes for green and scalable synthesis of graphene nanoribbons
Source: Sci Rep. 2016 Mar 7;6:22755. doi: 10.1038/srep22755 (PMC4780102; doi:10.1038/srep22755)
Supplement: Supplementary Information [file srep22755-s1.doc]

Intercalation-assisted longitudinal unzipping of carbon nanotubes for

green and scalable synthesis of graphene nanoribbons

Supplementary information

Yan-Sheng Li, Jia-Liang Liao,Shan-Yu Wang, and Wei-Hung Chang*

Department of Chemical Engineering, National Taiwan University of Science and Technology, Taipei, 10607.

* To whom correspondence should be addressed:[whchiang@mail.ntust.edu.tw](mailto:whchiang@mail.ntust.edu.tw) (W.-H. Chiang)

**CNT synthesis**

The CNTs used in the present study were synthesized using a catalytic chemical vapor deposition (CVD). Details of the CNT growth process were similar to that previously described [^1^](#_ENREF_1). In brief, Fe films (8.2 nm thickness) and an alumina (Al_2_O_3_) support layer (40 nm thickness) sputtered onto 2 cm × 2 cm polished silicon (Si) substrates with a silicon dioxide (SiO_2_) layer of 600 nm were used as the catalyst films for CNT growth. The average nominal thickness of ten as-deposited Fe films were characterized by ex situ AFM (Veeco Dimension 3100). The CNT were synthesized at one atmospheric pressure in a 3 inch quartz tube furnace with two process steps, including catalyst particle formation and CNT growth. For a typical catalyst particle formation experiment, we first flowed 200 sccm (sccm denotes standard cubic centimeter per minute at 1 atm helium (He) and 1800 sccm hydrogen (H_2_) for 15 minutes while ramping the temperature from room temperature to 810 °C, then keep same gas flow rates for 15 minutes to anneal the catalyst particles. Then CNT growth began for 10 minutes using a water-assisted CVD process at 810 °C with the gas mixture of 100 sccm ethylene (C_2_H_4_) and 900 sccm H_2_, and 100 ppm water vapor as the carbon precursor and the catalyst preserver and enhancer, respectively. Water vapor of 100 ppm was supplied by passing 1000 sccm He carrier gas through a water bubbler with deionized (DI) water at STP (STP denotes standard condition for temperature and pressure, NIST version) condition. Water vapor concentration was monitored by a single-channel moisture meter (General Electric, MMS 35-211-1-100) coupled with a moisture probe (General Electric, M2LR) installed before the CVD reactor. All gas flows were controlled by mass flow controllers that were carefully calibrated before experiments to precisely control the gas concentrations in the CVD reactor. Representative HR-TEM and SEM images of as-prepared CNT were shown in Figure S1 and S2, respectively, showing that the CNT was well prepared by atmospheric pressure CVD.

**GNR synthesis**

For the GNR synthesis, in a typical preparation^2^, 0.1g of MWCNTs was suspended in a different amount of concentrated H_2_SO_4_ and 1g KNO_3_ and then stirred at 300 rpm for 2 hrs using a magnetic stirrer until a visually homogeneous black solution formed. Detailed conditions are summarized in Table 1. Then KMnO_4_ with different amounts was slowly added to the solution and further stirred for 2 hrs at room temperature. After that, the temperature was gradually raised to different temperatures and maintained at that temperature for 2 hrs in water bath (IKA-HS7 digital). Detailed reaction conductions are summarized in Table 2. When the reaction was completed, the product was purified and dried with a series steps reported elsewhere.[^14^](#_ENREF_14) Briefly, each mixtures were removed from the heat source, allowed to cool to room temperature and poured into 350 g of ice containing 5 ml of 35% H_2_O_2_ each (to prevent precipitation of insoluble MnO_2_). The mixtures were then centrifuged (24500 rpm, 30 mins) to give crude GNRs solid (Beckman, Avanti J-25). The solid was removed and then bath-sonicated in 60 ml deionized (DI) water for 30 mins (IKA-HS7 digital). The material was bath-sonicated again by adding 30 ml HCl, and then the dispersion was centrifuged (24500 rpm, 30 mins). Furthermore, the collected solid was removed and then bath-sonicated in 60 ml ether for 30 mins. In the end, the purified GNRs were obtained by collecting the centrifuged (24500 rpm, 30 mins) solid.

**Characterizations**

The SEM images of raw MWCNTs and as-synthesized samples were performed on JEOL JSM-6500F (accelerating voltage =15 kV). Samples were prepared by pressing powders on the copper tape. The transmission electron microscopy images of raw MWCNTs and as produced GNRs were collected on Hitachi H-9500.Samples were prepared by dispersing aw MWCNTs and as-synthesized samples in ethanol and then drop onto 300 mesh holey lacy carbon grids on cupper support (Ted Pella, Inc.) at ambient condition. XPS was carried out on VG ESCA Scientific Theta Probe with pass energy 50 eV, 53° take off angle, and a 400 μm beam size. Samples were dispersed aw MWCNTs and as-synthesized samples in ethanol and formed thin film on silicon substrates at ambient condition. The XRD were obtained by BRUKER D2 PHASER- X-ray Powder Diffraction (Cu Kα, λ= 1.54 Å). The Raman spectra of pristine MWCNTs and as-pretreated samples were performed on JASCO NRS-5100 at 532nm excitation. The Fourier-transform infrared spectroscopy (FTIR) spectra of pristine CNTs and as-prepared samples were collected on Biorad FTS -3500.The samples were dispersed by ethanol and film casted on the potassium bromide (KBr) pallets. The thermogravimetric (TGA) data were collected by Perkin Elmer Diamond TG/DTA and was performed from room temperature to 800^o^C at the speed of 10 ^o^C min^-1^under argon (Ar) atmosphere. The atomic force microscopy (AFM) was carried out by Ardic instruments P-100. The samples were prepared by ethanol solution and spin coating on mica substrates.

**Figure S1** (a) SEM image of raw MWCNTs (scale bar = 500 nm). (b) TEM image of raw MWCNTs. (scale bar = 50 nm)

**Figure S2** Raman spectrum of raw MWCNTs.

**Figure S3** (a) G-band Raman shifts of pristine MWCNTs and different as-pretreated samples. (b) 2 theta diffraction angels of (002) planes of pristine MWCNTs and different as-pretreated samples. (c) C1s binding energies of pristine MWCNTs and different as-pretreated samples. The labels of intertube intercalation (shaded red) and intratube intercalation (shaded red) indicate the intertube intercalation and intratube intercalation of MWCNTs, respectively.

**Figure S4** Low magnification TEM images of (a) sample B and (b) sample D. (Scale bar = 1μm)

**Table S1** The summarized reaction conditions of MWCNT longitudinal unzipping and GNR yields. The amount of raw MWCNT is 0.1 g. Reaction time is 2h.

| Sample | H_2_SO_4_  (ml) | KNO_3_  (M) | K_2_CO_3_  (M) | K_2_S_2_O_8_  (M) | Temperature  (^o^C) | GNR yield form  XRD analysis (%) |
| --- | --- | --- | --- | --- | --- | --- |
| A | 10 | 1 | 0 | 0 | 30 | 56 |
| B | 10 | 1 | 0 | 0 | 70 | 100 |
| C | 100 | 0 | 0 | 0 | 70 | 100 |
| D | 10 | 0 | 0 | 0 | 70 | 80 |
| E | 0 | 1 | 0 | 0 | 70 | 0 |
| F | 10 | 0.5 | 0 | 0 | 70 | 92 |
| G | 10 | 2 | 0 | 0 | 70 | 80 |
| H | 10 | 0 | 0.5 | 0 | 70 | 85 |
| I | 10 | 0 | 1 | 0 | 70 | 83 |
| J | 10 | 0 | 2 | 0 | 70 | 72 |
| K | 10 | 0 | 0 | 0.5 | 70 | 74 |
| L | 10 | 0 | 0 | 1 | 70 | 25 |
| M | 10 | 0 | 0 | 2 | 70 | 12 |

**Reference**

1. Chiang, W.-H.; Futaba, D. N.; Yumura, M.; Hata, K., Growth Control of Single-Walled, Double-Walled, and Triple-Walled Carbon Nanotube Forests by a Priori Electrical Resistance Measurement of Catalyst Films. *Carbon* **2011**, *49*, 4368-4375.

2. Wang, C., Li, Y.-S., Jiang, J. & Chiang, W.-H. Controllable Tailoring Graphene Nanoribbons with Tunable Surface Functionalities: An Effective Strategy toward High-Performance Lithium-Ion Batteries. *ACS Appl. Mater. Interfaces* **7**, 17441-17449 (2015).
